# Neglected sexual dysfunction symptoms amongst chronic patients during routine consultations in rural clinics in the North West province

**DOI:** 10.4102/phcfm.v13i1.2850

**Published:** 2021-04-28

**Authors:** Deidre Pretorius, Ian D. Couper, Motlatso G. Mlambo

**Affiliations:** 1 Division of Family Medicine, School of Clinical Medicine, University of the Witwatersrand, Johannesburg, South Africa; 2 Ukwanda Centre for Rural Health, Faculty of Medicine and Health Sciences, Stellenbosch University, Cape Town, South Africa; 3 Department of Institutional Research and Business Intelligence, University of South Africa, Pretoria, South Africa

**Keywords:** sexual dysfunction, failure to care, diabetes, hypertension, sexual history, screening

## Abstract

**Background:**

Sexual dysfunction contributes to personal feelings of loss and despair and being a cause of exacerbated interpersonal conflict. Erectile dysfunction is also an early biomarker of cardiovascular disease. As doctors hardly ever ask about this problem, it is unknown how many patients presenting for routine consultations in primary care suffer from symptoms of sexual dysfunction.

**Aim:**

To develop an understanding of sexual history taking events, this study aimed to assess the proportion of patients living with symptoms of sexual dysfunction that could have been elicited or addressed during routine chronic illness consultations.

**Setting:**

The research was carried out in 10 primary care facilities in Dr Kenneth Kaunda Health District, the North West province, South Africa. This rural area is known for farming and mining activities.

**Methods:**

This study contributed to a broader research project with a focus on sexual history taking during a routine consultation. A sample of 151 consultations involving patients with chronic illnesses were selected to observe sexual history taking events. In this study, the patients involved in these consultations completed demographic and sexual dysfunction questionnaires (FSFI and IIEF) to establish the proportions of patients with sexual dysfunction symptoms.

**Results:**

A total of 81 women (78%) and 46 men (98%) were sexually active. A total of 91% of the women reported sexual dysfunction symptoms, whilst 98% of men had erectile dysfunction symptoms. The youngest patients to experience sexual dysfunction were a 19-year-old woman and a 26-year-old man. Patients expressed trust in their doctors and 91% of patients did not consider discussion of sexual matters with their doctors as too sensitive.

**Conclusion:**

Clinical guidelines, especially for chronic illness care, must include screening for sexual dysfunction as an essential element in the consultation. Clinical care of patients living with chronic disease cannot ignore sexual well-being, given the frequency of problems. A referral to a sexual medicine specialist, psychologist or social worker can address consequences of sexual dysfunction and improve relationships.

## Introduction

When considering sexual health, family planning and sexually transmitted infections are often the first things that come to mind and not sexual functioning. Yet, an estimated 14% of 12 097 patients in primary care are estimated to be coping with some form of sexual dysfunction.^1^ Chronic disease, medication side effects and age are commonly associated with sexual challenges and bring the clinician and patient together in the clinical encounter.^2,3,4,5^ Doctors should screen for reproductive health issues, sexual dysfunction and sexual risk behaviour when indicated.^6^ Often doctors will avoid such screening as the literature suggests that less than 70% of them feel uncomfortable to talk about sex.^7,8^

Patients also play a role in whether there is any discussion of sexual dysfunction during the consultation. Patient disclosure rates of sexual dysfunction during a consultation vary from 14% spontaneous disclosure to 20% if the clinician asks.^9,10,11^ Kingsberg et al. postulated that 68% of American men and women feared they would embarrass their clinician if they talked about sexual problems and 71% feared their complaints would be dismissed.^12^ An African study suggested that female patients were often unaware that sexual dysfunction was an illness or that they could get help from their doctors.^13^ By not discussing sexual dysfunction, patients are stuck with their understanding and misconceptions about the symptoms they experience and try to find their own remedies, some of which are potentially harmful.^14^ Not dealing with sexual challenges contributes to perceptions of infidelity, mistrust and intimate partner conflict and feelings of loss of masculinity, humiliation and despair.^15,16,17,18^

Sexual dysfunction is a common challenge for diabetic and hypertensive patients, frequently worsened by the lack of screening for and management thereof. Globally about 25% of people have arterial hypertension, which increases the risk of coronary artery disease and may precipitate sexual dysfunction.^19,20^ Although erectile dysfunction is not an independent cause of mortality, it is considered a biomarker for coronary artery disease.^21,22^ Hypertension has been linked to erectile dysfunction in men and lubrication and orgasmic challenges in women.^7,20^ Essential hypertension is commonly associated with female sexual dysfunction.^23^

Diabetes may also add to sexual distress. A meta-analysis of 145 studies showed erectile dysfunction is 3.5 times more likely to occur in men with diabetes when compared with a control group and overall 52.5% of diabetic males lived with erectile dysfunction.^24^ A recent study in South African primary care suggested that 97.3% of men with diabetes lived with erectile dysfunction.^25^ Sexual dysfunction in women with diabetes is often neglected, despite the fact that they suffer similar neuropathy, endocrine changes and vascular complications to men.^26^ A meta-analysis on sexual dysfunction and diabetes suggested that women living with diabetes were 2.02 times more likely to have sexual dysfunction than women in control groups.^27^ In Italy, of 595 women living with diabetes about 53.4% had sexual dysfunction with a significant difference (*p* = 0.001) between menopausal (63%) and non-menopausal women (41%).^28^ In a comparative study between 72 healthy and diabetic women in Turkey, the most common symptom found amongst the latter was lack of libido (77%), followed by diminished clitoral sensation (62.5%), orgasmic disorder (49%) and vaginal dryness (37.5%).^29^ A small study in Nigeria found that women with diabetes were less likely than a healthy control group to attempt sexual intercourse and experience arousal, pain and orgasm symptoms.^30^

No research has been carried out in primary care settings in South Africa to assess sexual dysfunction that can be potentially elicited during a recorded consultation. For this reason, the researcher aimed to observe sexual history taken during video-recorded routine consultations in rural primary care clinics. To improve rigour of the qualitative study, the researcher wanted to know in what proportion of these patients, sexual dysfunction was present.

The aim of the research was thus to determine, in a sample of patients with chronic illness participating in recorded consultations with primary care doctors, the proportion of patients living with symptoms of sexual dysfunction that could have been elicited or addressed during these consultations. The researcher also wanted to determine if the same patients expressed trust in their doctors and a willingness to discuss sensitive matters with them.

## Research methods and design

**FIGURE 1:**
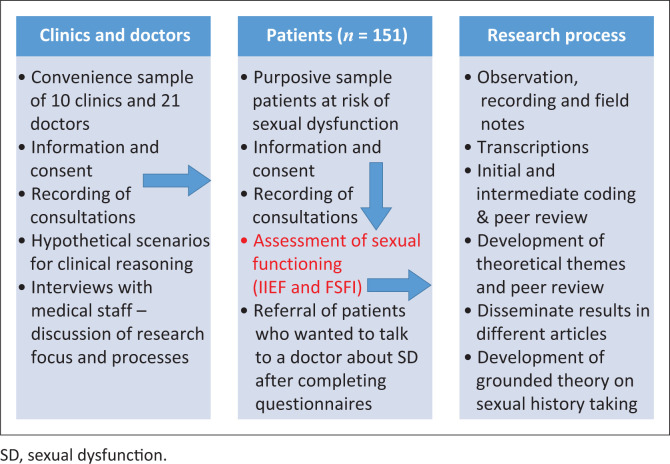
Assessment of sexual dysfunction as part of the broader study.

This study used a quantitative design to gather information, which would supplement broadly grounded research theory, to describe the sexual history taking process required to improve disclosure of sexual health issues during a clinical encounter with a clinician (Figure 1). The methodology of the overarching research project was reported in detail in another article.^31^

The study was carried out in the Dr Kenneth Kaunda Health District in the North West province. Most of the districts in this province can be considered rural. Mining and farming activities contribute to the economy of the area. Unemployment was a challenge for 37.2% of the local population at the time of the research.^32^

Consecutive adult patients living with diabetes and hypertension were purposefully selected based on the high risk of sexual dysfunction in these patients. The expected level of disclosure of sexual dysfunction was based on the literature was used to estimate the number of consultations that would be required to monitor 10 to 15 sexual history taking events A sample of 151 patients was thus selected for the overall research project; this sample was used as the cohort for the present study.

A trained research assistant recruited consecutive patients after they collected their files and had vital signs recorded. A total of 151 patients consented and participated. The research assistant informed the patients of the study and obtained consent in the patient’s home language both for video recording of the consultation and the completion of the questionnaire following the consultation. The patients completed a standardised questionnaire, namely Female Sexual Function Index (FSFI)^33^ for women and the International Index of Erectile Function (IIEF)^34^ for men assessing their sexual response for the previous 4 weeks. These questionnaires have not currently been validated in South Africa, but as they were designed based on Diagnostic and Statistical Manual of Mental Disorders (DSM) IV (now DSM-5) diagnostic criteria^35^ and the ICD10,^36^ both of which were considered appropriate to be used in South Africa. The questionnaires were administered in English, and the multilingual research assistant explained definitions of every category to the patients in their language of choice.

The researcher and research assistant collected all the data over 9 weeks and captured information nightly, checking with each other. The quantitative data were analysed using SAS (SAS Institute Inc., United States of America) Release 9.4. Descriptive statistics was used in the analysis applying a 95% confidence level. The age distribution passed the Shapiro–Wilk test for normality. Chi-square and Fisher’s exact test were carried out for association between demographic variables and sexual dysfunction.

### Ethical considerations

The study was approved by the Human Research Ethics Committee (Medical) of the University of the Witwatersrand (M160557), as well as the facility managers and the Directorate for Policy, Planning, Research, Monitoring and Evaluation of the Department of Health, the North West province, South Africa. Permission to use the questionnaires was obtained from Taylor and Francis online resource bank.

## Results

A total of 21 doctors were video-recorded during routine consultations of 151 patients and no sexual history taking for sexual dysfunction was observed in the recordings. These results were reported separately.^31^ The focus of this study was to report on the actual sexual dysfunction symptoms that these patients experienced, as well as their willingness and trust to discuss it with their doctors.

The distribution of participants was 104 (69%) women and 47 (31%) men. The study participants had a normal age distribution: men (aged 26 to 74) and women (aged 19 to 93). Women mentioned longer duration of relationships with a partner than men (Table 1). A total of 24 participants (16%) indicated that they were not sexually active (Table 1), for a variety of reasons. A total of 23 women were not sexually active: 11 (11%) abstained as they were afraid of contracting HIV, nine (6%) considered themselves too sick for sexual activity, two did not have opportunity to be sexually active and one abstained from sexual intercourse because of a history of rape. A 48-year-old man reported that he had not been sexually active for a long time because of erectile dysfunction. The participants who were not sexually active were excluded from the analysis of the sexual dysfunction questionnaires.

**TABLE 1:** Demographic data of participants.

Demographic criteria	Male patients (*n* = 47)		Female patients (*n* = 104)	
	*n*	%	*n*	%
**Age (years)**				
Age range	26–74	-	19–93	-
Mean	45	-	53	-
Median	49	-	55	-
**Marital status**				
Divorced	3	6	5	5
Estranged	-	-	1	1
Live together	5	11	5	5
Married	17	36	38	37
Separated	2	4	-	-
Single	14	30	29	28
Widowed	6	13	26	25
Not sexually active	-	-	-	-
Not sexually active	1	2	23	22
**Level of education**				
No formal education	-	-	1	1
Primary School (Gr 0–7)	19	40	42	40
Secondary school (Gr 8–12)	25	53	52	50
Tertiary training	3	6	9	9
**First language**				
Afrikaans	-	-	13	13
English	-	-	1	1
IsiXhosa	11	23	9	9
IsiZulu	1	2	2	2
Sesotho	6	13	18	17
Setswana	29	62	61	59
**Duration of relationship (years)**				
Relationship range	1–35	-	-	-
Mean	9.2	-	-	-
Median	5	-	-	-
Mode	2	-	-	-
Missing data	7	-	-	-

A total of 96 (64%) patients lived primarily with hypertension, while 11 (7%) patients lived with diabetes, and 44 (29%) patients had both diabetes and hypertension. Comorbidities included dyslipidaemia (29%), HIV (19%), epilepsy (7%), depression (3%) and tuberculosis (2%). Presenting complaints as recorded in the patients’ files were captured during data collection; some patients had more than one complaint, whereas 14 (9%) patients did not have any information recorded in that section (Table 2). Two patients had sexually related complaints, namely pain after intercourse and a sexually transmitted infection. Patients frequently used more than one medication that potentially could cause sexual dysfunction such as beta- and calcium-channel blockers and nearly all the patients used diuretics (Table 2).

**TABLE 2:** Presenting complaint and prescribed medication.

Presenting complaint	Male (*n* = 47)		Female (*n* = 81)	
	*n*	%	*n*	%
Repeat scripts/chronic follow-up	23	49	38	47
Pain (back, joints, abdominal, lower limbs)	12	26	15	19
Undifferentiated (dizziness, fatigue)	3	6	8	10
Results (histology, blood, etc.)	2	4	5	6
Administrative reasons	2	4	1	1
Sexually transmitted infections (STI)	1	2	-	-
Visual complaints	1	2	2	3
Other (deep vein thrombosis, breast mass, seizures)	1	2	9	11
Pain after intercourse	0	0	1	1
Stress	0	0	1	1
Missing data	4	8	7	9
Total number of complaints	49	-	87	-
**Medication used**				
Anticonvulsant†	4	9	1	1
Antipsychotic†	1	2	1	1
Antiretroviral therapy†	8	17	10	12
Benzodiazepine†	1	2	-	-
Beta-blocker†	10	21	20	25
Calcium-channel blocker†	11	23	33	41
Diuretics†	40	85	81	100
Lipid lowering agent†	7	15	31	38
Monoamine oxidase inhibitor†	1	2	2	2
Selective serotonin reuptake inhibitor†	1	2	3	-
Tricyclic antidepressant†	4	9	6	7
Acetylsalicylic acid	2	4	9	11
Angiotensin-converting enzyme inhibitors	11	23	27	33
Biguanides	8	17	23	28
Insulin	-	-	5	6
Sulphonylureas	1	2	6	7

STI, sexually transmitted infections; DVT, deep vein thrombosis

† , Medication potentially causing sexual dysfunction.^4^

### Female sexual dysfunction

A total of eight (5%) female patients disclosed spontaneously that their partners suffered sexual dysfunction, which affected their expression of sexuality. Of the 81 sexually active women, 76 (94%) women had FSFI scores < 26.55,^36^ which are suggestive of overall sexual dysfunction. A total of 73 (90%) women lived with low desire symptoms and only five (6%) considered themselves satisfied with their expression of sexuality. All the women had scores suggestive of challenges with lubrication and orgasm and 83% reported pain symptoms.

### Male sexual dysfunction

According to the IIEF results, 36 (78%) male patients met all the criteria for sexual dysfunction and 11 (24%) were satisfied with their overall sexual health (Table 3). A total of 14 (30%) participants experienced severe dysfunction in overall satisfaction, and the same number experienced intercourse dissatisfaction (Table 3). Only one man (2%) reported to have normal erectile function and one (2%) intercourse satisfaction. A total of 30 (64%) men lived with orgasmic challenges (Table 3).

**TABLE 3:** International Index of Erectile Function individual domain frequencies and range of male sexual dysfunction symptoms.

Degree of sexual adequacy for the domain†	Overall satisfaction		Erectile function		Orgasmic function		Sexual desire		Intercourse satisfaction	
	*n*	%	*n*	%	*n*	%	*n*	%	*n*	%
No sexual dysfunction	11	24	1	2	2	4	5	11	1	2
Mild sexual dysfunction	4	9	11	24	2	4	12	26	6	13
Mild to moderate challenges in sexual dysfunction	10	22	17	37	6	13	10	22	6	13
Moderate sexual dysfunction	7	15	7	15	6	13	13	28	19	41
Severe sexual dysfunction	14	30	10	22	30	65	6	13	14	30
Total number of participants	46	100	46	100	46	100	46	100	46	100

† Explanation of categories in relationship to responses:^34^ Severe sexual dysfunction (SD), Almost always or always; Moderate SD, Most times (much more than half the time); Mild to moderate SD, Sometimes (about half the time); Mild SD, A few times (much less than half the time); No SD, Almost never.

### Overall sexual dysfunction for men and woman and age

**FIGURE 2:**
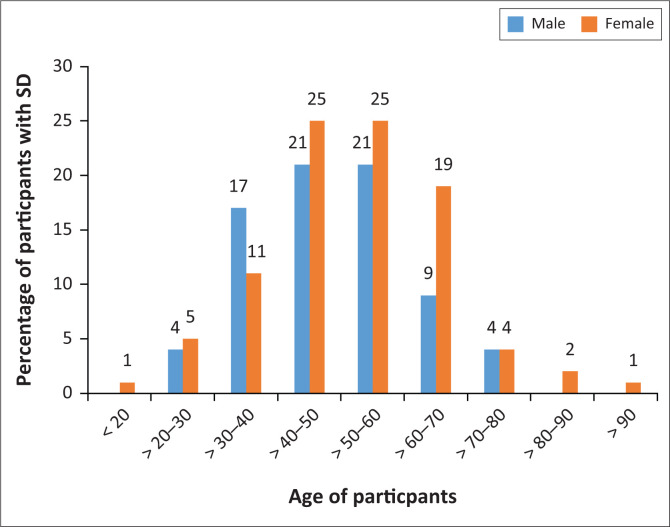
Percentage of male and female sexual dysfunction based on age category.

In terms of overall sexual dysfunction, most women (71%) experienced sexual dysfunction between the age of 41 and 70 years, unlike male sexual dysfunction that was more prominent between the age of 31 and 50 years (Figure 2). There was no statistical association between male or female sexual dysfunction and age, marital status, medication and living with diabetes or hypertension in this study.

### Perceptions of patients on disclosure of sexual dysfunction

The demographic questionnaire provided information on the patients’ perceptions of discussing sensitive matters such as sexual challenges. Answers to a question exploring whether patients would disclose sexual dysfunction to their doctors reflected that the majority of patient participants (77%) trusted their doctors all the time and only one patient did not trust doctors at all. A total of 85% of the participants considered sexual well-being as a general health matter and 91% did not perceive discussing sex with a clinician as a sensitive topic. A majority (74%) believed a clinician had to know about intimacy challenges.

## Discussion

It was important to know what proportion of this purposive sample of patients lived with symptoms of sexual dysfunction that could have been elicited or addressed during the recorded consultations. It was expected, as it was a high-risk patient sample, that history taking for sexual dysfunction would be observed.

The proportion of participants with sexual dysfunction symptoms in this study was high. For women the proportion with sexual dysfunction symptoms (94%) was considerably higher than a questionnaire-based study of 1219 women in Brazil where 49% of women had at least one sexual dysfunction symptom and women with diabetes presented with low sexual desire and orgasmic disorder.^37^ A study in Jordan found that 59.6% of women older than 50 years with diabetes (*n* = 613) had sexual dysfunction versus 45.6% of their comparison group of non-diabetic women (*n* = 524).^38^ The Jordan study also found that desire, arousal, lubrication and orgasm challenges were more prominent in older diabetic women, whilst our data showed that all ages presented lubrication, orgasm and pain challenges associated with both diabetes and hypertension.^38^ Similar to our study, a meta-analysis of 31 studies on sexual dysfunction in women with hypertension found that there was no statistical association between sexual dysfunction in women and medication such as beta-blockers, angiotensin-converting enzyme inhibitors, angiotensin receptor blockers, calcium channel blockers and diuretics.^39^

As causes of sexual dysfunction are complex, the high proportion of sexual dysfunction cannot be attributed only to disease and medication. Psychosocial factors certainly also have a role. The high prevalence of domestic violence in South Africa as either the cause or consequence of sexual dysfunction cannot be ignored. Interpersonal conflict is often associated with low sexual desire and sexual dysfunction.^40^ Socio-economic challenges such as homelessness, unemployment and even a lower educational level have been shown to be statistically associated with increased sexual dysfunction.^27,37,41^ Although this research did not elicit employment data, the research setting is neither an affluent area nor are there many job opportunities. Nearly half of the participants had no schooling or only primary schooling, which can influence the level of understanding and help-seeking for sexual dysfunction.^42,43^ Cultural values and expectations can also contribute to how the patients perceived and expressed their sexual functioning or challenges.^44,45^

A total of 77% of male participants lived with more than one symptoms of sexual dysfunction. Erectile dysfunction and orgasmic challenges were prominent in this study; the result of erectile dysfunction symptoms in this study was in line with a recent study in Gauteng province, South Africa, where 97% of the men attending a community health centre presented with erectile dysfunction.^25^ As erectile dysfunction is considered a biomarker for coronary artery disease,^22^ the failure to enquire about this raises questions about the identification and management of end organ damage of these patients in primary care clinics. Apart from medical issues, the psychological impact of not attending to the sexual functioning needs of the patient is immense as sexuality is often perceived as penis-driven and the loss of functioning thereof signals loss of manhood and its associated status.^18^ Intimate relationships may even suffer as the partner may perceive it as a lack of interest because of infidelity.^17^

This study provided insights into how early in adulthood men and women experienced symptoms suggestive of sexual dysfunction. For some women in this study sexual dysfunction already presented in late 20s and it was present in some men younger than 25 years old. Therefore, doctors should be cautioned to screen regularly for such problems.^46^ Unfortunately, the majority of studies on female sexual dysfunction usually include the older than 35 year group^46^ so it is difficult to say that the findings in this study for early adulthood sexual dysfunction are an outlier. We do however know that problems arising from childbirth can contribute to sexual dysfunction.^47^ As we did not elicit childbirth, it may be a reason other than diabetes or hypertension that younger women presented with sexual dysfunction. For both men and women, substance abuse must also be considered for sexual dysfunction symptoms.^48^

Patients in this study indicated that they trusted their doctors and did not consider sexual well-being as too sensitive a topic to discuss. It was not a matter of trust or sensitivity that prevented them from sharing their sexual challenges with doctors. With this big proportion of patients reporting symptoms in various degrees, the question was, if the doctors did not ask, why did the patients not simply tell them? Reviewing the literature suggests that sometimes patients stop asking for help because doctors avoid discussing sensitive matters.^6,12,49,50^

The patient cohort was selected for the theoretical prevalence of sexual dysfunction because of their diseases and the medication. Sexual dysfunction was assessed as part of the triangulation of data in the bigger qualitative study and therefore results are limited to the research setting. The strength of the study lies in the fact that it supplements research findings related to better understanding of sexual history taking during a consultation. For the first time we have recorded consultation findings with evidence of sexual dysfunction linked to those consultations and have demonstrated the disjuncture between levels of sexual dysfunction and extent of sexual history taking. It is observed that sexual functioning is complex and not only influenced by disease and medication but also psychological, trauma and social factors, which were not assessed. These data however give us a glimpse into the sexual challenges that patients in primary care present with, which are hardly ever discussed during a consultation.

From an ethical perspective, failing to screen for symptoms of sexual dysfunction not only prevented doctors from uncovering and thus exploring other pathologies, such as arterial disease, substance abuse or depression, but it also deprived patients of help that could have changed the quality of their sexual health and well-being.^51^ The South African standard treatment guidelines for both diabetes^52^ and hypertension^53^ and the Practical Approach to Care Kit (PACK)^54^ clinical decision support tool do not explicitly recommend screening for sexual dysfunction. However, if primary care, and even more family medicine, aims to render comprehensive healthcare, it must surely include screening for sexual dysfunction. Failing to screen for sexual dysfunction is not only a failure of history taking, but it is also a failure to care for patients and the problems they are living with. Despite the fact that psychosocial management of health and illness or preventative care are often not operationalised in the definition of quality and standards,^55^ and that doctors might feel they lack the knowledge and skills for the task, there remains a professional duty to provide care for the patient. The ideal would be if primary care doctors and family physicians are trained to initiate sexual history taking for sexual dysfunction. The role of the family physician in promoting the sexual health agenda in primary care must be explored in future research. At the very least, identification of these problems can secure a referral to an appropriate member of the healthcare team to address sexual dysfunction.^56^ A referral to doctors with special interest in psychologist or clinical social worker who are trained in sexual health can address some causes and most consequences of sexual dysfunction to improve relationships. Sexual dysfunction screening is thus not only a learned skill; it involves a paradigm shift from conducting ‘just another routine consultation’, to focusing on offering comprehensive clinical care.

Patients living with sexual dysfunction present every day somewhere in a healthcare facility in South Africa only to go home to live in emotional and sexual humiliation and despair. Clinical reasoning around patients living with chronic disease should not exclude sexual well-being – it is not a luxury, but rather a health necessity.
